# Healthcare resource utilisation and costs of agitation in people with dementia living in care homes in England - The Managing Agitation and Raising QUality of LifE in Dementia (MARQUE) study

**DOI:** 10.1371/journal.pone.0211953

**Published:** 2019-02-26

**Authors:** Monica Panca, Gill Livingston, Julie Barber, Claudia Cooper, Francesca La Frenais, Louise Marston, Sian Cousins, Rachael M. Hunter

**Affiliations:** 1 Research Department of Primary Care & Population Health and Priment Clinical Trials Unit, Institute of Epidemiology & Health Care, University College London, London, United Kingdom; 2 Department of Old Age Psychiatry, Division of Psychiatry, University College London, London, United Kingdom; 3 Department of Statistical Science, University College London, London, United Kingdom; Cardiff University, UNITED KINGDOM

## Abstract

**Background:**

People with dementia living in care homes often experience clinically significant agitation; however, little is known about its economic impact.

**Objective:**

To calculate the cost of agitation in people with dementia living in care homes.

**Methods:**

We used the baseline data from 1,424 residents with dementia living in care homes (part of **M**anaging **A**gitation and **R**aising **QU**ality of lif**E** in dementia (MARQUE) study) that had Cohen-Mansfield Agitation Inventory (CMAI) scores recorded. We investigated the relationship between residents’ health and social care costs and severity of agitation based on the CMAI total score. In addition, we assessed resource utilisation and compared costs of residents with and without clinically significant symptoms of agitation using the CMAI over and above the cost of the care home.

**Results:**

Agitation defined by the CMAI was a significant predictor of costs. On average, a one-point increase in the CMAI will lead to a 0.5 percentage points (cost ratio 1.005, 95%CI 1.001 to 1.010) increase in the annual costs. The excess annual cost associated with agitation per resident with dementia was £1,125.35. This suggests that, on average, agitation accounts for 44% of the annual health and social care costs of dementia in people living in care homes.

**Conclusion:**

Agitation in people with dementia living in care homes contributes significantly to the overall costs increasing as the level of agitation increases. Residents with the highest level of agitation cost nearly twice as much as those with the lowest levels of agitation, suggesting that effective strategies to reduce agitation are likely to be cost-effective in this setting.

## Introduction

It is estimated that 46.8 million people worldwide were living with dementia in 2015 with a total cost to society of US$818 billion. Given current population trends and that prevalence of dementia increases with age, the number of people with dementia worldwide is projected to triple to 131.5 million in 2050 [1].

In the UK, the estimated number of people living with dementia was over 800,000 in 2014 with a total cost to society of £26.3 billion. It is projected that the number of people with dementia in the UK will be over 1 million by 2025 and over 2 million by 2051 [2].

Dementia is characterised by progressive cognitive disability, a high prevalence of neuropsychiatric symptoms such as agitation, depression and psychosis, reduced quality of life, increased caregiver burden and costs of care [3, 4]. Symptoms of agitation comprise restlessness, pacing, shouting and verbal and physical aggression [5].

Agitation contributes to breakdown of care at home leading to care home placement [6] and this increases costs of care [7, 8]. It is estimated that more than half of people with dementia living in care homes have symptoms of agitation [9–12].

Although agitation in people living with dementia in their own homes is known to increase demand on health and social care services [13] and add to the family carer stress [14, 15], little is known about its economic impact [7]. A recent study [16] assessing the additional societal cost of care of agitation (based on Neuropsychiatric Inventory Questionnaire (NPI-Q) associated with dementia in 8 European countries, including England (mixed community and institutional long-term care) concluded that 53% of the total cost (total monthly mean cost differences due to agitation, 561€) was attributable to the institutional care cost and this was driven by higher outpatient cost.

We therefore aimed to compare the costs for care home residents with different levels of agitation measured by the Cohen-Mansfield Agitation Inventory (CMAI). In a sensitivity analysis we repeated the analysis using the Neuropsychiatric Inventory (NPI) agitation scale. In addition, we calculated the cost of agitation (over and above the cost of the care home) in residents with and without clinically significant symptoms of agitation using the CMAI as well as the excess costs associated with agitation.

## Methods

### Participants / Data source and study population

This study is part of the **M**anaging **A**gitation and **R**aising **QU**ality of lif**E** in dementia (MARQUE) study.

We have reported the recruitment strategy in detail elsewhere [12].

Care home managers agreed to care home inclusion, provided a staff list and identified residents with clinical diagnosis of dementia. For residents without a dementia diagnosis, managers completed the Noticeable Problems Checklist (NPC) [15, 17] to identify probable dementia. The NPC is a six-item questionnaire covering memory, basic self-care, orientation, naming familiar people and ability to follow conversations, which has been used by non-clinicians and which has been validated against clinical diagnosis. Eligible participants were all residents with an existing dementia diagnosis or those who screened positive for dementia, and their family and care home staff.

Staff asked residents, whom they judged to have decisional competence for consent, if researchers could approach them; they were asked for written informed consent to the study. Consultees were asked to make this decision for residents who lacked capacity in line with the Mental Capacity Act (2005). For all other residents, the staff tried to contact the next-of-kin (to participate in family carer interviews) and asked if the researchers could contact them. Participating staff and relatives gave written informed consent. A paid carer working closely with a resident and a family member was asked to rate proxy measures for the resident. Trained research assistants conducted interviews using semi-structured questionnaires.

Residents with dementia were recruited from personal care (residential) or nursing care homes across England between 13 January 2014 and 12 December 2015. Both types of care home provide accommodation, meals and help with personal care needs from staff 24 hours a day; however, nursing care homes also have registered nurses on duty at all times who can provide care for people with more complex needs and those who need regular nursing interventions.

### Measures

We report the ratings provided by interviewing staff from the following questionnaires.

#### Agitation

***Cohen-Mansfield Agitation Inventory (CMAI)*** is a caregivers’ rating questionnaire consisting of 29 agitated behaviours, each rated on a 7-point scale of frequency with which they manifest physically aggressive, physically non-aggressive and verbally agitated behaviours with 1 meaning “never” and 7 meaning “several times per hour”. CMAI systematically assesses agitation in people with dementia with construct validity and reliability to be used in care homes [4, 18]. A total CMAI score is obtained by summing all the individual items, giving a range from 29 to 203. A total score of >45 is usually regarded as clinically significant agitation [19].

***The Neuropsychiatric Inventory (NPI)*** assesses dementia-related behavioural symptoms examining 12 domains of behavioural functioning including agitation [20, 21]. Behaviours are rated in terms of frequency and severity and the product of these forms a score for each domain. Each domain scores between 0 and 12 giving nine possible values (0, 1, 2, 3, 4, 6, 8, 9, 12), including no symptoms (= 0) with higher scores meaning increasing severity (NPI agitation score). A score of ≥4 in any domain is usually regarded as clinically significant [22] A summed score (total NPI score) can also be calculated for total neuropsychiatric symptoms.

#### Healthcare resource use and costs

***Client Service Receipt Inventory (CSRI)*** [23]: we collected health care resources for the previous 4 months from a detailed resource use questionnaire, amended for use with older people in care homes.

This incorporated information on health and social care service use: primary care, community health or emergency services contacts (e.g., general practitioner (GP), practice nurse, specialist nurse, night nurse, and community psychiatric/ mental health team), accident and emergency attendances, outpatient contacts, inpatient admissions, social care contacts (e.g., social worker, extra carer), community based services contacts (e.g., chiropodist, optician, dentist), other professionals contacts (e.g., geriatrician, neurologist, psychiatrist).

All recorded medical prescriptions were aggregated for each resident, with the number of individual doses prescribed. Net ingredient costs per dose, based on the electronic British National Formulary (BNF) [24] and National Health Service (NHS) Electronic Drug Tariff [25] were applied to these dose records, in order to arrive at a total cost of prescriptions issued.

We included the NHS and personal and social services perspective in our costing analysis as it is the recommended costing in economic evaluations in the UK [26].

Costs were estimated by multiplying the number of units used for each relevant resource factor with the corresponding unit cost (S1 Table) from available sources [27, 28] at 2014/15 UK pounds (£).

Four-month costs were estimated for each resident by adding up total primary care, accident and emergency, outpatient, inpatient, social care and community services, other medical professional contacts and prescriptions costs.

The 4-month costs were multiplied by 3 to create annual (12-month) figures.

### Analysis

Descriptive data are presented as means and standard deviations (SD) and, where appropriate, median and interquartile range (IQR) for continuous outcomes and numbers and percentages (%) for categorical outcomes.

We calculated annual mean costs by CMAI scores (≤45 and >45). We also considered the relationship between annual total costs and continuous CMAI total scores. The cost data was highly skewed and therefore we used a generalised linear model (GLM). We considered GLM models with different link functions and distributions and chose the gamma family with logarithmic link based on graphical inspection of the residuals and the Akaike information criterion [29]. We present the exponential of the GLM model coefficients in the form of a cost ratio.

The dependent variable was the total costs and the independent variable the measure of agitation (CMAI total scores). We adjusted for demographic characteristics and other potential confounders that may influence the extent to which healthcare resources are used by this population. Covariates included in the model were age, sex, dementia severity (Clinical Dementia Rating (CDR score)) and care home type ((nursing, residential care or both, dementia registered (registered to care for people with dementia) or dementia specialist (have a dedicated dementia specialist and carers specially trained to provide tailored dementia care)).

Similar analyses with agitation defined by the total NPI scores in place of CMAI scores were conducted.

Analyses were performed using Stata version 14.1[30].

### Excess costs associated with agitation

We combined the adjusted annual costs per resident and percentages of residents at different levels of agitation based on CMAI score to calculate the annual expected costs per resident with dementia. We then subtracted the adjusted annual costs per residents with no clinical significant agitation (CMAI score ≤45) to estimate the mean excess costs associated with agitation.

## Results

### Demographic characteristics

1,489 care home residents with dementia were recruited to the study [12] (S1 File). Of the 1,483 residents with baseline data available, 1,424 residents had CMAI scores recorded; 40% of residents had CMAI scores >45 indicating clinically significant symptoms.

Demographic characteristics of residents with and without clinically significant agitation are presented in Table 1. The groups are similar in terms of the sociodemographic and other characteristics studied.

**Table 1:** Demographic characteristics by CMAI scores.

Characteristics		CMAI ≤45 (n = 855) N (%) or otherwise stated	CMAI >45 (n = 569) N (%) or otherwise stated	p-value
**Sex**	**Male**	246 (29%)	195 (34%)	0.03
	**Female**	609 (71%)	374 (66%)	
**Mean age (years)**		85.5 (8.4)	84.5 (8.5)	0.03
**Age category**	**≤60**	9 (1%)	7 (1%)	0.02
	**61–70**	40 (5%)	33 (6%)	
	**71–80**	154 (18%)	112 (20%)	
	**81–90**	393 (46%)	274 (48%)	
	**≥91**	245 (29%)	133 (23%)	
	**Missing**	14 (2%)	10 (2%)	
**Ethnicity**	**White British**	763 (89%)	485 (85%)	<0.001
	**White Irish**	24 (3%)	19 (3%)	
	**White other**	27 (3%)	20 (4%)	
	**Chinese**	0 (0%)	2 (0.4%)	
	**Black British, Caribbean, African**	18 (2%)	14 (2%)	
	**Asian or Asian British, Indian, Pakistani, Bangladeshi**	5 (1%)	8 (1%)	
	**Other**	9 (1%)	19 (3%)	
	**Missing**	9 (1%)	2 (0.4%)	
**Care home type**	**Nursing care home**	121 (14%)	91 (16%)	0.09
	**Personal care (residential) home**	307 (36%)	220 (39%)	
	**Nursing & personal care home**	427 (50%)	258 (45%)	
**Living in a dementia registered care home**		764 (89%)	535 (94%)	<0.001
**Living in a dementia specialist care home**		345 (40%)	273 (48%)	<0.001
**Dementia severity**	**Very mild**	98 (11%)	15 (3%)	0.01
	**Mild**	203 (24%)	102 (18%)	
	**Moderate**	255 (30%)	212 (37%)	
	**Severe**	296 (35%)	239 (42%)	
	**Missing**	3 (<1%)	1 (<1%)	
**CMAI scores**	**Mean (SD)**	35 (5)	64 (17)	<0.001
	**Median (IQR)**	34 (30, 39)	60 (51, 73)	
**NPI agitation scores**		324 (38%)	481 (85%)	
	**Mean (SD)**	3 (2)	4 (3)	0.4
	**Median (IQR)**	3 (1, 4)	4 (3, 8)	

CMAI = Cohen-Mansfield Agitation Inventory; NPI = Neuropsychiatric Inventory scale; SD = standard deviation; IQR = interquartile range; N = number.

### Healthcare resource use and costs

The unadjusted annual mean costs per resident with CMAI scores ≤45 and >45 were £2,410.21 (95% CI £2,158.74 to £2,661.67) and £2,800.66 (95%CI £2,451.74 to £3,149.58), respectively. The total costs in both groups was similar and was driven by healthcare resource utilisation at different settings except social care contacts, and prescriptions. The difference in costs between groups was not statistically significant (Table 2).

**Table 2:** Annual mean costs per resident by CMAI scores (£, 2014/15 UK).

Resource use	CMAI ≤45		CMAI >45		Difference
	N	Mean (95%CI)	N	Mean (95%CI)	Mean (95%CI)
**Overnight inpatient stay**	64	£10,761.23 (£9,808.52 to £11,713.95)	52	£11,054.83 (£9,904.20 to £12,205.46)	£293.60 (-£1,170.95 to £1,758.15)
**Outpatient contacts**	182	£ 431.04 (£393.91 to £468.18)	116	£398.33 (£363.70 to £432.96)	-£32.72 (-£86.64 to £21.21)
**Accident and Emergency contacts**	82	£ 439.46 (£408.87 o £470.05)	78	£467.08 (£427.03 to £507.12)	£27.61 (-£22.08 to £77.31)
**Primary care, community health or emergency contacts**	690	£765.88 (£685.28 to £846.47)	483	£802.76 (£699.24 to £906.29)	£36.89 (-£92.52 to £166.29)
**Social care contacts**	120	£166.43 (£127.17 to £205.68)	79	£274.52 (£56.36 to £492.68)	£ 108.09 (-£73.53 to £289.72)
**Community based service contacts**	521	£ 420.24 (£350.47 to £490.00)	354	£431.43 (£358.60 to £504.26)	£11.19 (-£92.44 to £114.83)
**Other medical professionals contacts**	201	£1,110.43 (£846.75 to £1,374.10)	159	£1,092.87 (£804.97 to £1,380.77)	-£17.56 (-£407.98 to £372.86)
**Prescriptions**	838	£312.81 (£276.23 to £349.39)	547	£360.82 (£316.66 to £404.99)	£48.01 (-£9.59 to £105.62)
**Total cost**	853	**£2,410.21** **(£2,158.74 to £2,661.67)**	568	**£2,800.66** **(£2,451.74 to £3,149.58)**	**£ 390.45** **(-£28.72 to £809.63)**

CMAI = Cohen-Mansfield Agitation Inventory; CI = confidence interval; N = number

The results of the regression model are presented in Table 3. Agitation defined by CMAI scores was a significant predictor of annual mean costs indicating that, on average, a one-point increase in the CMAI total scores will lead to a 0.5 percentage points (cost ratio 1.005, 95%CI 1.001 to 1.010) increase in the annual costs.

**Table 3:** Impact of agitation (by CMAI scores) and other factors on annual mean costs (£, 2014/15 UK), N = 1,396.

Covariates	Cost ratio	(95% CI)
**CMAI scores**	1.005	(1.001–1.010)
**Age (years)**	1.002	(0.991–1.013)
**Female**	0.942	(0.785–1.131)
**Dementia severity**		
*Very mild*	Reference	
*Mild*	0.786	(0.561–1.101)
*Moderate*	0.888	(0.641–1.231)
*Severe*	0.915	(0.663–1.261)
**Care home type**		
*Nursing care home*	Reference	
*Personal care (residential) home*	1.403	(1.088–1.810)
*Nursing & personal care home*	1.180	(0.924–1.508)
**Dementia registered care home**	0.802	(0.593–1.086)
**Dementia specialist care home**	1.027	(0.865–1.220)

CMAI = Cohen-Mansfield Agitation Inventory; CI = confidence interval; N = number

In addition, living in residential care homes (without taking into account the care home costs) was a significant predictor of additional annual costs (cost ratio 1.403; 95%CI 1.088 to 1.810).

The CMAI scores in our sample ranged from 29 to 137. We calculated the annual mean costs with levels of CMAI scores that range from 29 to 139 for all observations at intervals of 10 (other variables held at their observed values).

With other variables at their observed values and CMAI scores held at 29 for all observations, the annual mean cost was £2,321.69 (95%CI £2,057.76 to £2,585.63); when CMAI scores = 139 the annual mean cost was £4,218.18 (95%CI £2,394.01 to £6,042.34) (S2 Table).

Sensitivity analyses with agitation defined by total NPI scores (used as a linear term rather than categorical indicators) showed a non-significant increase of 2.8 percentage point (cost ratio 1.028; 95%CI 0.993 to 1.065) in annual costs for one-point increase in the total NPI scores (S3 Table).

### Excess costs associated with agitation

The adjusted annual expected cost per resident with dementia based on the percentage with each CMAI score was £2,564.39. The adjusted annual expected cost of residents with no clinical significant agitation was £1,439.04 (S4 Table). This results in an excess annual cost of agitation of £1,125.35 accounting for 44% of the health and social care costs of dementia in care homes.

## Discussion

### Main findings

This is the first study to consider the cost of agitation in residents living in care homes, using data from MARQUE cross-sectional study, the biggest care home study to date. Previously the cost of agitation has been considered in the main because it contributed to admission to care homes. We found a significant increase in healthcare costs as the CMAI scores increase (cost ratio 1.005, 95%CI 1.001 to 1.010).

Although, scoring the answers on the CMAI scale may not indicate a clinical difference between consecutive CMAI scores (e.g., 49 and 50), there are other factors that may affect the CMAI score and contribute to the incremental cost. One study [31] reported that for every 1-point increase on the CMAI, there is a 3% increase in the likelihood of caregivers rating patient pain (p = 0.049); another study supports this finding [32]. The management of pain could conduct to the difference in costs.

In contrast, the accompanying paper of this study [12] reported that staff did not consider that agitation might be a manifestation of pain. Therefore, other factors could have had an impact on the cost. Higher agitation levels were significantly associated with lower staff and family ratings of the resident’s quality of life, and with prescription of antipsychotics and hypnotics: agitation was associated with lower quality of life (regression coefficient (rc) = −0.53, 95%CI = −0.61 to −0.46) and antipsychotic use (rc = 6.45, 95%CI = 3.98 to 8.91).

On average, agitation accounts for 44% of the annual health and social care costs of dementia in people living in care homes. Sensitivity analyses with agitation defined by NPI scores showed an increase in annual mean costs, although not statistically significant.

People with dementia and agitation are more likely to be admitted to care homes as the family carer becomes unable to look after them. According to the PayingForCare report for 2016/17 [33], people are expected to pay on average more for nursing care homes than residential care homes per year in the UK (£43,700 vs £31,200) and that depends whether they need nursing care or not. Our study shows that even within residential care homes which provide 24 hour care, residents with agitation use more health and social care resources as they require additional nursing or dementia care to their personal care. Although nursing care homes are more expensive than residential care homes, overall cost may be similar for both types of care homes due to additional resource utilisation in residential care homes. However, this additional resource utilisation is paid for by the health services, whereas up-front costs for nursing care home placements are paid for by the individual and/or social care.

### Strengths and weaknesses

This is the largest study of residents with dementia-related agitation in care homes to date. It covered varied care homes throughout England. The dataset provided detailed information on residents’ characteristics, including severity of dementia as well as use of healthcare services. The information is relatively recently collected which shows current practice in healthcare provided for people with dementia.

Our analysis showed that agitation (using CMAI scale) is associated with increase in healthcare costs in people with dementia. However, this is an observational study and therefore, this may not be causative and there could be other unobserved confounding factors that are contributing to both costs and agitation, although we have controlled for demographic features and dementia severity. We did not include the costs of family care although families continue to contribute when members of the family move to a care home; these data were not collected.

We used the CMAI total score in our analysis and have not controlled for the impact of particular behaviours/clusters of behaviours (e.g., aggressive vs non-aggressive) on cost as well as associated comorbidities. CMAI scores based on particular types of behaviours might be associated with higher costs. However, our study did not aim to look into particular behaviours on the CMAI scale but on the CMAI score as a total.

### Comparison with other studies

To our knowledge, no previous large study has assessed the impact of dementia-related agitation on health and social care costs in a care home setting. There are two studies that assessed the costs of behavioural symptoms particularly in Alzheimer’s disease–AD–(type of dementia that causes problems with memory, thinking and behaviour) in the UK, using the NPI agitation scale in community or mixed community and care home setting. Morris *et al*. [8] concluded that, on average, agitation symptoms accounted for 12% of the health and social care costs of AD. Gustavsson *et al*. [34] studied people residing at home or in care homes and showed no significant effect of agitation on costs for the residents with AD in care homes in the UK, Spain and Sweden (N = 70–118). In the United States only, one point increase in the NPI-severity had significant effect on costs (increase by 1.6%).

Similarly, our secondary analyses using total NPI scores found a non-significant increase in health care costs with agitation severity possibly because of the much narrower definition of agitation used in the NPI compared to CMAI.

### Implications

Health and social care costs in people with dementia increase with level of agitation. Investment should be made into developing interventions to reduce agitation given not only the potential positive impact on quality of life of residents, care home staff and carers; these are likely to represent value for money if they reduce the consumption of healthcare resources. A systematic review [35] of the clinical effectiveness and cost-effectiveness of non-pharmacological interventions for reducing agitation in adults with dementia evaluated 11 interventions with impact on the CMAI. The incremental cost per unit reduction in CMAI score ranged from £162 to £3,480 for activities, £4 for music therapy, from £24 to £143 for sensory interventions and from £6 to £62 for training and supervising paid caregivers in person-centred care or communication skills, behavioural management training or dementia care mapping.

## Ethics

This study reports the MARQUE longitudinal care home study baseline findings. It received ethics permission from NRES Committee London- Harrow 14/LO/0034.

## Supporting information

S1 Table Unit costs. Costs were estimated by multiplying the number of units used for each relevant resource factor with the corresponding unit cost from available sources at 2014/15 UK pounds (£).
(DOCX)

S2 Table Annual mean costs per resident by CMAI scores (2014/15 UK£) at each 10 incremental point, N = 1,396. Annual mean costs increase as the level of agitation measured by CMAI increases.
(DOCX)

S3 Table Impact of agitation (by total NPI scores) and other factors on annual mean costs (2014/15 UK£), N = 818. Sensitivity analyses with agitation defined by total NPI scores showed a non-significant increase of 2.8 percentage point (cost ratio 1.028; 95%CI 0.993 to 1.065) in annual costs for one-point increase in the NPI scores.
(DOCX)

S4 Table Calculation of excess costs associated with agitation in people with dementia in care homes. The excess annual cost of agitation was £1,125.35 accounting for 44% of the health and social care costs of dementia in care homes.
(DOCX)

S1 File MARQUE baseline flow diagram of recruited residents. Flow diagram of recruitment of residents for MARQUE study
(DOCX)
