# Supplementary material for: Healthcare resource utilisation and costs of agitation in people with dementia living in care homes in England - The Managing Agitation and Raising QUality of LifE in Dementia (MARQUE) study
Source: PLoS One. 2019 Feb 26;14(2):e0211953. doi: 10.1371/journal.pone.0211953 (PMC6391021; doi:10.1371/journal.pone.0211953)
Supplement: S1 Table — Costs were estimated by multiplying the number of units used for each relevant resource factor with the corresponding unit cost from available sources at 2014/15 UK pounds (£). (DOCX) [file pone.0211953.s001.docx]

**S1 Table. Unit costs**

| **Cost component** | **Unit cost*** | **Unit** | **Reference** |
| --- | --- | --- | --- |
| **Overnight inpatient stay** | | | |
| *Acute psychiatric ward inpatient stay* | 352 | Per day | 28 |
| *General medical elective/planned inpatient stay* | 447 | Per day | 28 |
| *General medical intensive care/high dependency unit inpatient stay* | 1043 | Per day | 28 |
| *General medical non-elective/unplanned inpatient stay* | 366 | Per day | 28 |
| *Geriatric ward inpatient stay* | 322 | Per day | 28 |
| *Psychiatric rehabilitation ward inpatient stay* | 325 | Per day | 28 |
| **Accident and emergency attendance** | 141 | Per contact | 28 |
| **Community-based health care services** | | | |
| *Audiology service* | 30 | Per contact | 28 |
| *Community matron / nurse* | 79 | Per contact | 28 |
| *Community physiotherapist* | 52 | Per contact | 28 |
| *Community psychiatric nurse/ community mental health nurse* | 67 | Per hour | 27 |
| *Day care* | 35 | Per visit | 27 |
| *Dentist* | 88 | Per hour | 27 |
| *Dementia intensive support worker/ dementia outreach* | 51 | Per hour | 27 |
| *Deprivation of liberty safeguards assessor* | 1393 | Per assessment | 27 |
| *Dietician/ nutritionist* | 83 | Per contact | 28 |
| *Falls clinic* | 250 | Per contact | 28 |
| *General practitioner* | 38 | Per care home consultation | 27 |
| *Learning disability support worker* | 81 | Per hour | 27 |
| *Night sitter/ carer* | 20 | Per hour | 27 |
| *Nurse assessor (continuing care)* | 42 | Per hour | 27 |
| *Occupational therapist* | 73 | Per contact | 28 |
| *Older persons community team worker* | 13 | Per hour | 27 |
| *Optician* | 89 | Per contact | 28 |
| *Paramedic (emergency services)* | 180 | Per contact | 28 |
| *Podiatrist/ chiropodist* | 40 | Per contact | 28 |
| *Practice nurse* | 47 | Per contact | 27 |
| *Speech and language therapist* | 87 | Per contact | 28 |
| *Specialist nurse (anticoagulant clinic)* | 44 | Per contact | 28 |
| *Specialist nurse (breast care)* | 44 | Per contact | 28 |
| *Specialist nurse (cardiac)* | 72 | Per contact | 28 |
| *Specialist nurse (continence)* | 81 | Per contact | 28 |
| *Specialist nurse (dementia)* | 42 | Per hour | 27 |
| *Specialist nurse (diabetes)* | 68 | Per contact | 28 |
| *Specialist nurse (enteral feeding)* | 79 | Per contact | 28 |
| *Specialist nurse (epilepsy)* | 81 | Per hour | 27 |
| *Specialist nurse (palliative care)* | 79 | Per contact | 28 |
| *Specialist nurse (Parkinson’s)* | 77 | Per contact | 28 |
| *Specialist nurse (respiratory)* | 78 | Per contact | 28 |
| *Specialist nurse (tissue viability)* | 55 | Per contact | 28 |
| *Specialist nurse (urology)* | 81 | Per contact | 28 |
| *Wheelchair service* | 125 | Per assessment | 28 |
| **Social care** | | | |
| *Social worker or care manager* | 40 | Per hour | 27 |
| *Home care/home help worker* | 24 | Per hour | 27 |
| *Meals on wheels* | 3.62 | Per meal | 27 |
| **Outpatient and other professionals contacts** | | | |
| *Day patient procedure/test* | 134 | Per procedure | 28 |
| *General medical* | 114 | Per contact | 28 |
| *Geriatrician* | 226 | Per contact | 28 |
| *Memory clinic* | 424 | Per hour | 27 |
| *Neurologist* | 176 | Per contact | 28 |
| *Oncologist* | 159 | Per contact | 28 |
| *Ophthalmologist* | 92 | Per contact | 28 |
| *Plastic surgeon* | 93 | Per contact | 28 |
| *Psychiatrist* | 106 | Per contact | 28 |
| *Psychologist* | 201 | Per contact | 28 |
| *Radiologist* | 42 | Per contact | 28 |
| *Urologist* | 99 | Per contact | 28 |

*(2014/15 UK£)
