# Supplementary material for: Healthcare resource utilisation and costs of agitation in people with dementia living in care homes in England - The Managing Agitation and Raising QUality of LifE in Dementia (MARQUE) study
Source: PLoS One. 2019 Feb 26;14(2):e0211953. doi: 10.1371/journal.pone.0211953 (PMC6391021; doi:10.1371/journal.pone.0211953)
Supplement: S2 Table — Annual mean costs increase as the level of agitation measured by CMAI increases. (DOCX) [file pone.0211953.s002.docx]

**S2 Table. Predicted annual mean costs per resident by CMAI scores (£, 2014/15 UK) at each 10 incremental point, N=1,396**

| **CMAI scores** | **Annual mean cost (95%CI)** |
| --- | --- |
| **29** | £2,321.69 (£2,057.76 to £2,585.63) |
| **39** | £2,451.20 (£2,235.95 to £2,666.45) |
| **49** | £2,587.94 (£2,376.00 to £2,799.88) |
| **59** | £2,732.30 (£2,460.30 to £3,004.30) |
| **69** | £2,884.71 (£2,505.06 to £3,264.37) |
| **79** | £3,045.63 (£2,527.52 to £3,563.74) |
| **89** | £3,215.52 (£2,535.18 to £3,895.87) |
| **99** | £3,394.90 (£2,530.69 to £4,259.10) |
| **109** | £3,584.27 (£2,514.72 to £4,653.82) |
| **119** | £3,784.21 (£2,487.09 to £5,081.33) |
| **129** | £3,995.31 (£2,447.16 to £5,543.46) |
| **139** | £4,218.18 (£2,394.01 to £6,042.34) |

CMAI=Cohen-Mansfield Agitation Inventory scale; CI=confidence interval; N=number

*Regression model controls for demographic variables, dementia severity and care home facilities
