# Supplementary material for: Healthcare resource utilisation and costs of agitation in people with dementia living in care homes in England - The Managing Agitation and Raising QUality of LifE in Dementia (MARQUE) study
Source: PLoS One. 2019 Feb 26;14(2):e0211953. doi: 10.1371/journal.pone.0211953 (PMC6391021; doi:10.1371/journal.pone.0211953)
Supplement: S3 Table — Sensitivity analyses with agitation defined by total NPI scores showed a non-significant increase of 2.8 percentage point (cost ratio 1.028; 95%CI 0.993 to 1.065) in annual costs for one-point increase in the NPI scores. (DOCX) [file pone.0211953.s003.docx]

**S3 Table. Impact of agitation (by total NPI scores) and other factors on annual mean costs (2014/15 UK£), N=818**

| **Covariates** | **Cost ratio** | **(95% CI)** |
| --- | --- | --- |
|  |  |  |
| **Total NPI scores** | 1.028 | (0.993-1.065) |
| **Age (years)** | 1.003 | (0.990-1.016) |
| **Female** | 1.034 | (0.826-1.294) |
| **Dementia severity** |  | |
| *Very mild* | Reference |  |
| *Mild* | 1.092 | (0.594-2.006) |
| *Moderate* | 1.186 | (0.661-2.127) |
| *Severe* | 1.229 | (0.685-2.205) |
| **Care home type** |  | |
| *Nursing care home* | Reference |  |
| *Personal care (residential)home* | 1.248 | (0.905-1.723) |
| *Nursing & personal care home* | 1.043 | (0.765-1.422) |
| **Dementia registered care home** | 0.975 | (0.650-1.463) |
| **Dementia specialist care home** | 1.040 | (0.841-1.287) |

NPI=Neuropsychiatric Inventory; CI=confidence interval; N=number
