# Supplementary material for: Healthcare resource utilisation and costs of agitation in people with dementia living in care homes in England - The Managing Agitation and Raising QUality of LifE in Dementia (MARQUE) study
Source: PLoS One. 2019 Feb 26;14(2):e0211953. doi: 10.1371/journal.pone.0211953 (PMC6391021; doi:10.1371/journal.pone.0211953)
Supplement: S4 Table — The excess annual cost of agitation was £1,125.35 accounting for 44% of the health and social care costs of dementia in care homes. (DOCX) [file pone.0211953.s004.docx]

**S4 Table. Calculation of excess costs associated with agitation in people with dementia in care homes**

| **CMAI score** | **Percent observations**  **N=1,424**  **(A)** | **Adjusted mean annual costs per resident**  **(B)** |
| --- | --- | --- |
| 29 | 14.68 | £ 2,321.69 |
| 30 | 2.67 | £ 2,334.33 |
| 31 | 4.07 | £ 2,347.03 |
| 32 | 2.74 | £ 2,359.81 |
| 33 | 4.35 | £ 2,372.65 |
| 34 | 3.93 | £ 2,385.57 |
| 35 | 3.23 | £ 2,398.55 |
| 36 | 3.30 | £ 2,411.61 |
| 37 | 3.37 | £ 2,424.73 |
| 38 | 2.25 | £ 2,437.93 |
| 39 | 2.46 | £ 2,451.20 |
| 40 | 2.18 | £ 2,464.54 |
| 41 | 2.46 | £ 2,477.96 |
| 42 | 2.18 | £ 2,491.45 |
| 43 | 2.25 | £ 2,505.01 |
| 44 | 2.32 | £ 2,518.64 |
| 45 | 1.62 | £ 2,532.35 |
| 46 | 1.90 | £ 2,546.13 |
| 47 | 1.62 | £ 2,559.99 |
| 48 | 1.69 | £ 2,573.93 |
| 49 | 2.32 | £ 2,587.94 |
| 50 | 1.76 | £ 2,602.02 |
| 51 | 1.83 | £ 2,616.19 |
| 52 | 1.40 | £ 2,630.43 |
| 53 | 1.54 | £ 2,644.74 |
| 54 | 0.49 | £ 2,659.14 |
| 55 | 1.05 | £ 2,673.61 |
| 56 | 1.05 | £ 2,688.16 |
| 57 | 0.98 | £ 2,702.80 |
| 58 | 0.91 | £ 2,717.51 |
| 59 | 1.05 | £ 2,732.30 |
| 60 | 0.70 | £ 2,747.17 |
| 61 | 0.77 | £ 2,762.12 |
| 62 | 0.84 | £ 2,777.16 |
| 63 | 1.12 | £ 2,792.27 |
| 64 | 1.19 | £ 2,807.47 |
| 65 | 1.54 | £ 2,822.75 |
| 66 | 0.63 | £ 2,838.12 |
| 67 | 0.84 | £ 2,853.57 |
| 68 | 0.42 | £ 2,869.10 |
| 69 | 0.84 | £ 2,884.71 |
| 70 | 0.42 | £ 2,900.42 |
| 71 | 0.63 | £ 2,916.20 |
| 72 | 0.28 | £ 2,932.08 |
| 73 | 0.56 | £ 2,948.03 |
| 74 | 0.77 | £ 2,964.08 |
| 75 | 0.49 | £ 2,980.21 |
| 76 | 0.63 | £ 2,996.44 |
| 77 | 0.63 | £ 3,012.74 |
| 78 | 0.28 | £ 3,029.14 |
| 79 | 0.21 | £ 3,045.63 |
| 80 | 0.49 | £ 3,062.21 |
| 81 | 0.70 | £ 3,078.88 |
| 82 | 0.42 | £ 3,095.63 |
| 83 | 0.42 | £ 3,112.48 |
| 84 | 0.14 | £ 3,129.43 |
| 85 | 0.28 | £ 3,146.46 |
| 86 | 0.49 | £ 3,163.58 |
| 87 | 0.28 | £ 3,180.80 |
| 88 | 0.21 | £ 3,198.12 |
| 89 | 0.35 | £ 3,215.52 |
| 92 | 0.14 | £ 3,268.32 |
| 93 | 0.07 | £ 3,286.11 |
| 94 | 0.07 | £ 3,303.99 |
| 95 | 0.21 | £ 3,321.98 |
| 97 | 0.07 | £ 3,358.24 |
| 98 | 0.14 | £ 3,376.52 |
| 100 | 0.14 | £ 3,413.37 |
| 101 | 0.21 | £ 3,431.95 |
| 103 | 0.14 | £ 3,469.41 |
| 104 | 0.07 | £ 3,488.30 |
| 105 | 0.07 | £ 3,507.29 |
| 106 | 0.07 | £ 3,526.38 |
| 107 | 0.14 | £ 3,545.57 |
| 108 | 0.07 | £ 3,564.87 |
| 110 | 0.07 | £ 3,603.78 |
| 111 | 0.14 | £ 3,623.40 |
| 113 | 0.07 | £ 3,662.95 |
| 115 | 0.07 | £ 3,702.93 |
| 118 | 0.14 | £ 3,763.73 |
| 119 | 0.07 | £ 3,784.21 |
| 120 | 0.14 | £ 3,804.81 |
| 121 | 0.07 | £ 3,825.52 |
| 125 | 0.07 | £ 3,909.49 |
| 127 | 0.07 | £ 3,952.17 |
| 134 | 0.14 | £ 4,105.23 |
| 137 | 0.07 | £ 4,172.63 |

CMAI=Cohen-Mansfield Agitation Inventory scale; N=number

Annual expected cost per person with dementia based on percent with each CMAI score = (sum of (column A [CMAI score from 29 to 137]*column B [CMAI score from 29 to 137])) =£2,564.39

Annual cost per resident with dementia and no clinically significant agitation = (sum of (column A [CMAI score from 29 to 45]*column B [CMAI score from 29 to 45])) =£1,439.04

Mean annual excess costs associated with agitation=£2,564.39-£1,439.04=£1,125.35
