# Supplementary material for: Healthcare resource utilisation and costs of agitation in people with dementia living in care homes in England - The Managing Agitation and Raising QUality of LifE in Dementia (MARQUE) study
Source: PLoS One. 2019 Feb 26;14(2):e0211953. doi: 10.1371/journal.pone.0211953 (PMC6391021; doi:10.1371/journal.pone.0211953)
Supplement: S1 File — Flow diagram of recruitment of residents for MARQUE study (DOCX) [file pone.0211953.s005.docx]

**S1 File. MARQUE baseline flow diagram of recruited residents**

Residents assessed for eligibility (*n*=3542)

Eligible residents (*n*=3053)

Not approached for consent (n=228)

Died (*n*=172)

In hospital (*n*=12)

Left home (*n*=44)

Losses after consent (*n*=6)

Resident died (*n*=5)

Resident left care home (*n*=1)

Did not consent (n=1336)

Refused (*n*=772)

Unable to contact family (*n*=497)

Always sleeping (*n*=5)

Unwell (*n*=10)

Potential distress (*n*=6)

No available consultee (*n*=20)

Other (*n*=26)

Any baseline data available (*n*=1483)

Consented (*n*=1489)

Approached or consent (*n*=2825)

Not eligible as no dementia (*n*=489)
